# Racial–Geographic Disparity in Lipid Management in Veterans with Type 2 Diabetes: A 10-Year Retrospective Cohort Study

**DOI:** 10.1089/heq.2019.0071

**Published:** 2019-09-23

**Authors:** Elizabeth A. Brown, Ralph C. Ward, Erin Weeda, David J. Taber, Robert Neal Axon, Mulugeta Gebregziabher

**Affiliations:** ^1^ Department of Health Professions, College of Health Professions, Medical University of South Carolina, Charleston, South Carolina.; ^2^ Department of Public Health Sciences, College of Medicine, Medical University of South Carolina, Charleston, South Carolina.; ^3^ Health Equity and Rural Outreach Innovation Center, Ralph H. Johnson Department of Veterans Affairs Medical Center, Charleston, South Carolina.; ^4^ Department of SCCP Clinical Pharmacy and Outcome Sciences—MUSC Campus, College of Pharmacy, Medical University of South Carolina, Charleston, South Carolina.; ^5^ Department of Surgery and College of Medicine, Medical University of South Carolina, Charleston, South Carolina.; ^6^ Department of Medicine, College of Medicine, Medical University of South Carolina, Charleston, South Carolina.

**Keywords:** veterans, diabetes, race/ethnicity, rural residence, disparities

## Abstract

**Purpose:** The prevalence of diabetes in U.S. veterans (20.5%) is nearly three times that of the general population. Minority veterans have higher rates of diabetes compared with their counterparts and urban/rural residence is also associated with uncontrolled cholesterol. However, the interplay between urban/rural residence and race/ethnicity on cholesterol control is unclear.

**Methods:** Veterans Health Administration Corporate Data Warehouse and Centers for Medicare and Medicaid data were used to create unique dataset and perform longitudinal study of veterans with type 2 diabetes from 2006 to 2016. Logistic regression was used to model the association between low-density lipoprotein (LDL) control and the primary exposures (race/ethnicity and location of residence) after adjusting for all measured covariates, including the interaction between location of residence and race/ethnicity.

**Results:** There was a significant interaction between race/ethnicity and rural residence. Rural non-Hispanic Black (NHB) veterans had higher odds for LDL >100 mg/dL (odds ratio [OR]=1.70, 95% confidence interval [CI] 1.50–1.60) and for LDL >70 mg/dL (OR=1.59, 95% CI 1.53–1.64) compared with urban non-Hispanic White (NHW) veterans. Similarly, compared with urban NHW, urban NHB veterans had higher odds of LDL >100 mg/dL (OR=1.45, 95% CI 1.43–1.47) and LDL >70 mg/dL (OR=1.36, 95% CI 1.34–1.38).

**Conclusion:** This study highlights health disparities for veterans with type 2 diabetes. Future research is needed to evaluate interventions for mitigating these disparities in cholesterol management among veterans with diabetes.

## Introduction

Racial and ethnic minorities in the United States have a substantially higher prevalence of type 2 diabetes compared with their nonminority counterparts, and the prevalence of diagnosed diabetes in U.S. veterans (20.5%) is nearly three times that of the U.S. general population.^1^ Furthermore, minority veterans have higher rates of diabetes and heart disease compared with non-Hispanic white (NHW) veterans.^2^ Several evidence-based guidelines have been established to prevent cardiovascular disease (CVD) events, including controlling A1C, blood pressure, and low-density lipoprotein (LDL) cholesterol.^3–5^ However, based on a study of over 11,000 U.S. veterans, non-Hispanic Black (NHB) veterans had odds that were 1.4, 3.6, and 7.7 times greater for having 1, 2, or 3 uncontrolled risk factors, respectively, compared with NHW veterans.^6^

Lipid-lowering therapy is a key CVD risk reduction strategy. Yet, NHBs have a lower likelihood of receiving lipid-lowering medications.^7–9^ One study examining statin prescribing patterns in patients with diabetes reported NHW males had the highest proportion with statin treatment and had better LDL control compared with minority counterparts.^10^ Without proper treatment, providers and health care systems may see more patients with CVD events due to poor control, particularly minority patients. NHBs tend to have poor control of LDL compared with NHW.^7,10,11^ In 2017, researchers reported NHB women had the highest proportion of uncontrolled LDL among treated and untreated patients, whereas NHW men had greater LDL control compared with all other groups in the study (e.g., NHB women, NHB men, and NHW women).^10^ In a study of veterans with diabetes, NHBs also had lower odds of LDL control (odds ratio [OR] 0.66, 95% confidence interval [CI] 0.60–0.72) compared with NHW veterans.^11^

There is mixed evidence that a patient's geographical location may be associated with LDL outcomes. One study described differences in diabetes care between rural and urban practices in Alabama and reported fewer rural patients had good LDL control.^12^ Other studies found no such difference in LDL control by region^7^ or rural residence.^13^ In Southeastern states, there was no significant relationship between rural residence and LDL control; however, LDL tended to be higher in rural areas.^13^ Other researchers reported LDL control was actually higher in the stroke belt compared with areas outside of the stroke belt.^7^

To our knowledge, there is no long-term published data examining the effect of rural residence and racial/ethnic differences on LDL control in veterans with diabetes. Therefore, we sought to evaluate the impact of rural residence and race/ethnicity on LDL control in U.S. veterans with type 2 diabetes.

## Methods

### Data source

This was a retrospective cohort study of national clinical and administrative data in adult veterans with type 2 diabetes. This cohort had been formed for an earlier study,^14^ and we expanded the dataset to merge Medicare Part D data as described below. Multiple clinical and administrative files from the Veterans Health Administration (VHA) Corporate Data Warehouse (CDW) and Centers for Medicare and Medicaid (CMS) data were linked to create a unique dataset containing a large cohort of veterans with type 2 diabetes.

The Veterans Health Information Systems and Technology Architecture (VistA) was the primary source for the CDW data extracts, which included prescription data, diagnostic codes, laboratory values, and demographic information embedded in outpatient visit, outpatient pharmacy, and inpatient admission domains. Medicare Part A, B, and D data were linked to the Department of Veteran Affairs (VA) dataset. Our study's 2006–2016 timeframe is based on the Part D program's launch on January 1, 2006. The datasets were linked using patient scrambled social security numbers.

The study was approved by the Institutional Ethics Review Board of the Medical University of South Carolina (MUSC). The authors report no potential conflicts of interest relevant to this article. This article represents the views of the authors and not those of the MUSC or VHA.

### Study population

Inclusion criteria were: (1) veterans with type 2 diabetes (*n*=729,822) as defined by two or more ICD-9 codes for diabetes (250, 357.2, 362.0, and 366.41) during the 24 months before 2002 and again during 2002 with prescriptions for insulin or oral hypoglycemic agents in 2002 based on a previously validated algorithm^14,15^; (2) 65 years or older on January 1, 2006 (Medicare qualified). Veterans who met the study inclusion criteria were: identified and followed longitudinally from January 2006 until December 2016, loss to follow-up, or death.

### Exposure and covariates

The primary exposure variables were the combination of race/ethnicity and location of residence. Race/ethnicity was defined based on VA and CMS sources and was classified as NHW, NHB, Hispanic, and other, with NHW serving as the reference group. The term “NHB” is used to describe African American or black populations, and the term “NHW” is used to describe white populations to maintain consistent terminology. Location of residence (urban, rural) was based on Rural Urban Commuting Area (RUCA) codes, which were derived from the patient's zip code; urban location was coded as the reference.

We also controlled for several demographic, clinical, and socioeconomic variables. Age was treated as continuous and centered at a mean of 74.2 years. Gender was treated as nominal with males as the reference group. Smoking status was classified as smoker and nonsmoker (reference group). Marital status was classified as unmarried or married (reference group). Percentage service connectedness, representing the degree of disability related to military service, was dichotomized at 50%, with the <50% group serving as the references. Patients with service-connected diabetes and those with greater than 50% service-connected disability do not pay for medications in the VA system, whereas others are usually subject to a copay. Statin use was measured annually and categorized by the following groups: (1) no statin use (reference); (2) statin as regular dose; and (3) intense statin dose.^16^ Medical comorbidities were based on International Classification of Disease Clinical Modification (ICD-CM) 9 and 10 codes obtained from both VA and CMS. ICD-10 codes were applicable after October 1, 2015. ICD codes were summarized by the Elixhauser comorbidity index definition.^17^ We controlled for the following clinical variables: number of annual primary care visits (time-varying), major adverse cardiac events (acute coronary syndrome, atherosclerotic cerebrovascular disease, coronary heart disease, acute coronary syndrome), and mean annual A1C control (≤8%, >8%; time-varying). Finally, we also controlled for annual CMS-VHA dual utilization, splitting groups by >80% VHA, 50–80% VHA, and <50% VHA utilization, with the first group defined as the reference group.

Dual-use status was time varying over the study period, based on a patient's annual primary care visits and inpatient stays.

### Outcome

The primary outcomes were uncontrolled LDL cholesterol dichotomized at two cutoff values (>100 or >70 mg/dL). The reference for each scenario was controlled LDL (LDL <100 mg/dL) or (LDL <70 mg/dL).

### Statistical analyses

In preliminary analyses, crude associations were examined between LDL control and all measured covariates using chi-square tests for categorical variables and *t*-tests for continuous variables. Logistic regression was used to model the association between LDL control and the primary exposures (race/ethnicity and location of residence) after adjusting for all measured covariates, including the interaction between location of residence and race/ethnicity. ORs and associated 95% CIs were computed from univariate and an adjusted models using generalized estimating equation (GEE) type models with adjustment for clustering and repeated measures through the _RANDOM_ statement in PROC GLIMMIX of SAS 9.4. Our analysis assumes missing at random which we found to be reasonable given the many covariates involved and extent of missing data we have in the LDL outcome variable (30%). A GEE type logistic regression model with missing LDL as the outcome produced estimates (OR and 95% CI) through PROC GLIMMIX for examining which covariates were most associated with missingness. There were 28% and 30% missing values in A1C and LDL in the records, with 23% missing both A1C and LDL (Supplementary Table S1). A stronger predictor of missingness was A1C (OR=1.15, 1.14–1.17), which is adjusted for in the model. Residual analysis was used to assess goodness of fit. All analyses were conducted using SAS v. 9.4 (SAS Institute, Inc., Cary, NC).

## Results

Demographic characteristics of the population (*n*=729,822) from 2006 to 2016 are listed in Table 1. Most patients were from urban areas (58.6%), NHW (82.9%), male (98.8%), and married (59.8%). Most had ischemic heart disease (IHD; 66.2%). The mean age for the cohort was 74.2 years old with the all-cause mortality rate of 68.5%. A slightly higher percentage of patients from rural areas were smokers (15.2%) and had a history of major adverse cardiac events.

**Table 1.** Demographic and Clinical Characteristics, 2006–2016

Variable	Level	Location		Total
		Urban	Rural	
*N*^a^	No. (%)	427,766 (58.6%)	257,056 (41.4%)	729,822
Age	Mean (std)	74.4 (6.1)	73.8 (5.9)	74.2 (6.0)
Mortality rate	Before December 31, 2016	68.4	68.5	68.5
Sex	Male (%)	98.5	98.9	98.8
Race–ethnicity	Non-Hispanic white (%)	77.9	91.6	82.9
	Non-Hispanic black (%)	12.8	4.9	9.8
	Hispanic (%)	7.0	1.7	5.1
	Other race (%)	2.2	1.8	2.1
Marital status	Married (%)	58.3	62.2	59.8
Disability	>50% Service-related (%)	17.4	15.9	16.8
Smoking status	Smoker (%)	13.2	15.2	14.0
No. of Elixhauser comorbidities	Mean number per group (std)	7.8 (3.4)	8.1 (3.3)	7.9 (3.4)
No. of primary care visits	Mean per year (std)	4.5 (4.0)	4.3 (4.0)	4.4 (4.0)
Hemoglobin A1C	Percent ≥8%	14.1	14.5	14.2
Major adverse cardiac events (%)	Acute coronary syndrome	23.1	26.5	24.3
	Atherosclerotic cerebrovascular disease	20.0	22.8	20.6
	Coronary heart disease	64.5	69.1	66.2
	Peripheral artery disease	45.3	47.8	46.2
Statins prescription filled (%)^a^	None	18.2	16.8	17.7
	Low intensity statin	77.1	78.7	77.7
	High intensity statin	4.7	4.5	4.6
Dual VA-CMS utilization (%)^a^	>80% VA utilization	49.2	42.5	46.9
	50–80% VA utilization	20.7	25.8	22.5
	<50% VA utilization	30.1	31.7	30.6

^a^ Values based on average group membership 2006–2016.

CMS, Centers for Medicare and Medicaid; VA, Department of Veteran Affairs.

### LDL >100 mg/dL outcome

Table 2 provides results for sequential models. In the base model that included only time, location, and race/ethnicity, veterans with type 2 diabetes from rural areas had 17% higher odds of having LDL >100 mg/dL compared with those from urban areas, and NHB patients had 53% higher odds of having LDL >100 mg/dL. In the full model, increased odds of LDL >100 mg/dL were higher for the rural-NHB group (OR 1.70, 95% CI 1.65–1.75) and the urban-NHB group (OR 1.45, 95% CI 1.43–1.47), compared with the urban-NHW group. Patients prescribed statins, at either low/moderate or high-intensity doses, had significantly lower odds of LDL >100 mg/dL when compared with those not prescribed statins. However, this improvement was slightly less among those on a high-intensity statin: OR 0.53 (95% CI 0.52–0.54) for patients on high intensity versus OR 0.47 (95% CI 0.46–0.48) for those on low/moderate-intensity doses. Patients with an A1C >8% had 30% higher odds of having an LDL >100 mg/dL. Females had 80% higher odds of LDL >100 mg/dL, as compared with men.

**Table 2.** Sequential Models for the Odds of Poor Low-Density Lipoprotein Cholesterol Control (>100 mg/dL)

Variable	Level	Outcome: LDL >100		
		Base model	With race^*^location	Full model
Year	(per year)	0.94 (0.94–0.94)	0.94 (0.94–0.94)	0.95 (0.94–0.95)
Location of residence	Urban	Ref		
	Rural	1.17 (1.16–1.18)		
Race–ethnicity	Non-Hispanic white	Ref		
	Non-Hispanic black	1.53 (1.51–1.55)		
	Hispanic	1.21 (1.19–1.23)		
	Other race	1.1 (1.07–1.14)		
Race^*^location	Non-Hispanic white^*^urban		Ref	Ref
	Non-Hispanic white^*^rural		1.18 (1.17–1.19)	1.19 (1.18–1.2)
	Non-Hispanic black^*^urban		1.54 (1.52–1.56)	1.45 (1.43–1.47)
	Non-Hispanic black^*^rural		1.76 (1.71–1.82)	1.7 (1.65–1.75)
	Hispanic^*^urban		1.24 (1.21–1.26)	1.14 (1.11–1.16)
	Hispanic^*^rural		1.24 (1.17–1.3)	1.16 (1.1–1.23)
	Other race^*^urban		1.12 (1.08–1.16)	1.08 (1.04–1.12)
	Other race^*^rural		1.26 (1.2–1.34)	1.23 (1.17–1.3)
Sex	Male			Ref
	Female			1.83 (1.76–1.9)
Age	(Per year)			1 (1–1)
Marital status	Married			Ref
	Unmarried			1.1 (1.09–1.11)
Disability	>50% service-related			1 (0.99–1.01)
Smoking Status	Nonsmoker			Ref
	Smoker			1.03 (1.02–1.05)
A1C	≤8%			Ref
	>8%			1.27 (1.26–1.28)
Number primary care visits	Per year			1 (0.99–1)
Major adverse cardiac events	Acute coronary syndrome			1.11 (1.09–1.12)
	Atherosclerotic cerebrovascular disease			1.07 (1.06–1.08)
	Coronary heart disease			0.76 (0.75–0.77)
	Peripheral artery disease			0.99 (0.98–1)
Statins prescribed	No statins			Ref
	Statins at regular dose			0.47 (0.46–0.48)
	Intense statin dose			0.53 (0.52–0.54)
Dual VA-CMS status	>80% VA utilization			Ref
	50–80% VA utilization			1.03 (1.02–1.04)
	<50% VA utilization			0.98 (0.98–0.99)

The gray shading is to indicate that the terms corresponding to these rows are omitted.

ORs (95% CIs) for GEE models.

CI, confidence interval; GEE, generalized estimating equation; LDL, low-density lipoprotein; OR, odds ratio; Ref, reference category.

### LDL >70 mg/dL outcome

Table 3 provides results for sequential models. In the base model, veterans with type 2 diabetes from rural areas had 13% higher odds of having LDL >70 mg/dL compared with those from urban areas, and NHB patients had 41% higher odds of having LDL >70 mg/dL. In the full model, increased odds of LDL >70 mg/dL were again particularly seen for the rural-NHB group (OR 1.55, 95% CI 1.50–1.60) and the urban-NHB group (OR 1.36, 95% CI 1.34–1.38), both compared with the urban-NHW group. Patients prescribed statins at either regular or high-intensity doses had significantly lower odds of LDL >70, when compared with those not prescribed statins, with improvement again slightly lower for those on high-intensity doses. Patients with annual mean A1C >8% had 13% higher odds of having LDL >70. Females had 58% higher odds of LDL >70, as compared with men. Table 4 shows the odds ratios for the association between poor low-density lipoprotein cholesterol control (>70 mg/dL or >100 mg/dL) and Elixhauser comorbidity indicators. These indicators were added to the models displayed in Tables 2 and 3 to adjust for the confounding effect of comorbidities.

**Table 3.** Sequential Models for the Odds of Poor Low-Density Lipoprotein Cholesterol Control (>70 mg/dL)

Variable	Level	Outcome: LDL >70		
		Base model	With race^*^location	Full model
Year	(Per year)	0.92 (0.92–0.92)	0.92 (0.92–0.92)	0.92 (0.92–0.92)
Location of residence	Urban	Ref		
	Rural	1.13 (1.12–1.14)		
Race–ethnicity	Non-Hispanic white	Ref		
	Non-Hispanic black	1.41 (1.39–1.43)		
	Hispanic	1.14 (1.12–1.16)		
	Other race	1.04 (1.01–1.07)		
Race^*^location	Non-Hispanic white^*^urban		Ref	Ref
	Non-Hispanic white^*^rural		1.14 (1.13–1.15)	1.15 (1.14–1.16)
	Non-Hispanic black^*^urban		1.41 (1.39–1.43)	1.36 (1.34–1.38)
	Non-Hispanic black^*^rural		1.59 (1.53–1.64)	1.55 (1.5–1.6)
	Hispanic^*^urban		1.15 (1.13–1.17)	1.06 (1.04–1.08)
	Hispanic^*^rural		1.17 (1.11–1.23)	1.11 (1.05–1.16)
	Other race^*^urban		1.06 (1.02–1.09)	1.02 (0.98–1.05)
	Other race^*^rural		1.14 (1.08–1.2)	1.13 (1.07–1.19)
Sex	Male			Ref
	Female			1.58 (1.52–1.65)
Age	(Per year)			1 (1–1)
Marital status	Married			Ref
	Unmarried			1.05 (1.04–1.06)
Disability	>50% service-related			0.99 (0.98–1.01)
Smoking status	Nonsmoker			Ref
	Smoker			1 (0.98–1.01)
A1C	≤8%			Ref
	>8%			1.13 (1.12–1.14)
Number primary care visits	Per year			1 (1–1)
Major adverse cardiac events	Acute coronary syndrome			1.02 (1.01–1.03)
	Atherosclerotic cerebrovascular disease			1.04 (1.03–1.05)
	Coronary heart disease			0.75 (0.74–0.76)
	Peripheral artery disease			0.99 (0.98–0.99)
Statins prescribed	No statins			Ref
	Statins at regular dose			0.55 (0.55–0.56)
	Intense statin dose			0.57 (0.56–0.58)
Dual VA-CMS status	>80% VA utilization			Ref
	50–80% VA utilization			1.03 (1.02–1.04)
	<50% VA utilization			0.97 (0.96–0.98)

The gray shading is to indicate that the terms corresponding to these rows are omitted.

ORs (95% CIs) for GEE models.

**Table 4.** Odds Ratios for the Association Between Poor Low-Density Lipoprotein Cholesterol Control and Elixhauser Comorbidity Covariates (>70 mg/dL or >100mg/dL) from the Full Models in Tables 2 and 3

	LDL >100	LDL >70
Elix_aids	1.27 (1.14–1.41)	1.21 (1.09–1.35)
Elix_alcohol	1.15 (1.13–1.17)	1.11 (1.09–1.13)
Elix_anemdef	0.87 (0.86–0.88)	0.89 (0.88–0.9)
Elix_arth	1.13 (1.11–1.15)	1.09 (1.07–1.11)
Elix_bldloss	0.98 (0.96–1)	0.96 (0.94–0.97)
Elix_chf	0.95 (0.93–0.96)	0.89 (0.88–0.9)
Elix_chrnlung	1.05 (1.04–1.07)	1.05 (1.04–1.06)
Elix_coag	0.88 (0.87–0.9)	0.88 (0.87–0.89)
Elix_depres	1.12 (1.11–1.14)	1.09 (1.07–1.1)
Elix_drug	1.06 (1.03–1.1)	1.01 (0.98–1.03)
Elix_htn	1.03 (1.01–1.05)	1.05 (1.04–1.07)
Elix_htncx	1.02 (1.01–1.04)	1.02 (1.01–1.04)
Elix_hypothy	1.03 (1.01–1.04)	1.01 (0.99–1.02)
Elix_liver	1.05 (1.03–1.07)	0.98 (0.96–1)
Elix_lymph	1.01 (0.98–1.03)	0.96 (0.94–0.98)
Elix_lytes	1.01 (1–1.03)	0.99 (0.97–1)
Elix_mets	1.09 (1.07–1.11)	1.05 (1.03–1.06)
Elix_neuro	1.1 (1.09–1.12)	1.07 (1.05–1.08)
Elix_obese	0.96 (0.95–0.98)	0.98 (0.97–0.99)
Elix_para	1.1 (1.08–1.12)	1.04 (1.02–1.06)
Elix_psych	1.11 (1.09–1.13)	1.07 (1.06–1.09)
Elix_pulmcirc	1.02 (1–1.04)	1.01 (0.99–1.02)
Elix_renlfail	0.97 (0.96–0.99)	0.97 (0.96–0.99)
Elix_tumor	1.06 (1.04–1.07)	1.07 (1.05–1.08)
Elix_ulcer	0.99 (0.94–1.05)	1 (0.96–1.05)
Elix_valve	1.02 (1.01–1.04)	1.06 (1.05–1.07)
Elix_wghtloss	1.07 (1.05–1.08)	1.02 (1–1.03)

## Discussion

In this large national sample of older veterans with type 2 diabetes, NHB from rural areas were significantly less likely to have optimally controlled LDL levels, as defined as LDL below either 100 or 70 mg/dL. Although statin therapy was associated with lower odds of poor LDL control, this improvement was slightly smaller among those on high-intensity doses.

Earlier studies reported a racial disparity in LDL outcomes, including receiving LDL screening,^18,19^ receiving appropriate statin therapy,^19^ and achieving LDL control.^18,20^ Researchers compared veterans based on National Cholesterol Education Program (NCEP) guidelines and found that NHB veterans (compared with NHW) had a significantly lower rate of adherence to simvastatin (40.9% vs. 56.9%, *p*=0.0001), and NHB veterans also had a substantially higher mean LDL (138 mg/dL vs. 113 mg/dL).^20^ In another study, NHB patients with diabetes were less likely to receive statin therapy compared with their NHW counterparts.^19^ It is possible that fewer screenings, lower medication adherence, and lower likelihood of receiving statin therapy may lead to a higher LDL in NHBs; however, there are other factors that may increase poor LDL outcomes.

Differences in care by race/ethnicity, caused by implicit or explicit bias or based on differences in other factors, such as care coordination, may impact how patients view and receive advice from health care entities and medical providers. For example, minority patients may not completely trust medical advice from providers, which could inevitably affect management and treatment of various conditions, including high cholesterol. Historically, vulnerable populations (e.g., minorities, low-income, and religious groups) have experienced unfortunate events in health care and may have a certain level of mistrust in health care systems and providers. For NHBs, the Tuskegee Syphilis Study is one such event that may further heighten hesitation and fear when interacting with medical providers.^21^

Another factor that may explain the racial disparity in LDL control could be patient's education. Researchers used National Health and Nutrition Examination Survey (NHANES) data from 1999 to 2002 to examine the effect race and education had on diagnosis and management of high cholesterol.^22^ The study found participants with less than a high school education had a 2.5 times less likelihood to be screened for high cholesterol.^22^ Unfortunately, we did not have access to information on educational attainment in this analysis. According to the U.S. Census Bureau, in 2017, a higher proportion of NHW (compared with NHB) reported a higher education: bachelor's degree (23.8% vs. 15.1%), master's degree (10.5% vs. 7.0%), professional degree (1.8% vs. 0.7%), or doctoral degree (2.1% vs. 1.1%).^23^ These studies and national educational attainment data illustrate how patient demographics, particularly race/ethnicity and education, may impact screening, treatment, and control of high cholesterol in minority populations.

We should note that in a study examining veterans with IHD, researchers founds no such racial disparity in high cholesterol management when using NCEP guidelines.^24^ Chart reviews were done at five VA hospitals from August 1999 to January 2001. NHB veterans had a higher mean LDL than NHW veterans, but the difference was not significant between the two groups (118.2 mg/dL vs. 112.4 mg/dL, *p*=0.27). Yet, researchers discovered NHB veterans were significantly less likely to receive appropriate lipid-lowering medications compared with NHW veterans (46.2% vs. 59.6%, *p*=0.0003).^24^

In addition to race/ethnicity, rural residence was associated with greater odds of poor LDL control. Individuals and families in rural areas have a unique set of barriers compared with people living in more urban areas, particularly issues with access to care. People living in rural areas may live in medically underserved areas, travel further distance for specialty care, and may have less income due to minimal job opportunities. Across 8 southeastern U.S. states and 113 nonmetropolitan counties, a higher proportion of NHB rural residents (compared with NHW rural residents) had less than a high school education (23.4% vs. 15.7%), and a higher percentage of rural NHW (24.9%) completed college compared with rural NHBs (16.3%).^25^ Researchers found several notable findings: NHBs had significantly increased odds of (1) not getting needed care (OR 1.39); (2) having difficulty getting routine care (OR 1.67); (3) being uninsured (OR 1.92); (4) seeking emergency room care (OR 2.82); (5) having difficulty getting transportation (1.79); and (6) believing there were racial barriers are issues in accessing health care (OR 4.40).^25^ This study is important to our findings because it shows a racial and geographic (rural) disparity for accessing care, which could impact high cholesterol screening, treatment, and management; furthermore, the study highlights that even within the rural community, racial/ethnic minorities face increased odds for poor access to care.

In another study, researchers found that most Latinos from rural areas reported they did not have adequate access to affordable or nutritious food and had a higher likelihood of poor outcomes, including A1C >8% and LDL >100 mg/dL.^26^ This group also had significantly higher odds of poor medication adherence due to cost barriers (OR 2.49). In this case of rural Latinos, we see how rural residence, cost barriers, and access (or lack of) to healthy food options, can potentially impact clinical outcomes like A1C and LDL control.

Based on our study, race/ethnicity and rural residence are associated with increased odds of poor LDL control. However, other factors that rural minorities may encounter, particularly low education attainment, cost barriers, racism, travel barriers, and food insecurity, may negatively impact patient care-seeking behavior. For example, if rural minority patients have low health literacy, mistrust of providers, and cost or transportation barriers, they may be less likely to seek care, which may contribute to poor LDL control. This could in turn produce a higher rate of CVD events and mortality among these patients.

One possible approach to address patient behaviors and encourage self-efficacy in seeking care is peer navigation for people with diabetes. In one such study in rural parts of Alabama,^27^ researchers reported significantly improved outcomes for systolic blood pressure, body mass index (BMI), quality of life, and patient activation. While this trial did not have a significantly positive effect on LDL, it is important to consider such programs to encourage patients to seek care in their communities. It is also important to consider the time needed to instill trust and communication in the patient/provider relationship. This study lasted ∼15 months; however, it may take longer to see substantive changes in patient attitudes and behaviors.

It is important to note several study limitations. First, we were not able to examine several relevant factors affecting subjects' socioeconomic status (e.g., income, education, or employment status). Controlling for these factors may have affected our OR estimates for LDL control. Second, our study spans 10 years in clinical practice, and it is possible that unobserved health system factors (e.g., policy changes) or patient behaviors (e.g., patient care-seeking behavior or medication adherence) may impact findings. However, we did assess for time trends in prescribing patterns for statins, and these were not apparent. Third, because of our large sample size, we note that all interaction terms evaluated were statistically significant. While this was not surprising, we were struck by the magnitude of the interaction between NHB race and rural residence.

## Conclusion and Implications for Health Equity

Our results indicate race/ethnicity and urban/rural residence are associated with higher odds of poor LDL control in older veterans with diabetes. Various factors associated with race/ethnicity and rural residence (e.g., mistrust, minimal resources, low health literacy, issues with access to care) may lead to fewer minority veterans from rural areas being screened or appropriately treated for high cholesterol. Future research is needed to examine the impact of these observations on CVD events and mortality and cost to the VA system. Furthermore, research should explore and evaluate peer navigation program(s) that may improve clinical outcomes for veterans with chronic conditions.

## Disclaimer

This article represents the views of the authors and not those of the VHA HSR&D or MUSC.

## Authors' Contributions

The research idea was conceived by M.G. The analysis was done by R.C.W. and M.G. The first draft of the article was prepared by E.A.B. All coauthors participated substantially in the writing and critical review of the article. All authors approved the final version of the submitted article.

## Supplementary Material

Supplemental data

## Abbreviations Used

CDW: Corporate Data Warehouse
CI: confidence interval
CMS: Centers for Medicare and Medicaid
CVD: cardiovascular disease
GEE: generalized estimating equation
ICD-CM: International Classification of Disease Clinical Modification
IHD: ischemic heart disease
LDL: low-density lipoprotein
MUSC: Medical University of South Carolina
NCEP: National Cholesterol Education Program
NHB: non-Hispanic Black
NHW: non-Hispanic white
OR: odds ratio
VA: Department of Veteran Affairs
VHA: Veterans Health Administration
