# Supplementary material for: Racial–Geographic Disparity in Lipid Management in Veterans with Type 2 Diabetes: A 10-Year Retrospective Cohort Study
Source: Health Equity. 2019 Sep 23;3(1):472–9. doi: 10.1089/heq.2019.0071 (PMC6767165; doi:10.1089/heq.2019.0071)
Supplement: Supplemental data [file Supp_Table1.pdf]

Supplementary Data

Supplementary Table S1. Model for Predicting Missing Low-Density Lipoprotein Data

	Logit model with HbA1C	Logit Model without HbA1C
Year (per year)	1.09 (1.09, 1.09)	1.09 (1.09, 1.10)
Age (per year)	1.00 (1.00, 1.00)	1.00 (1.00, 1.00)
Elixhauser comorbidities[a]	1.01 (0.99, 1.02)	1.02 (1.02, 1.02)
Primary care visits (per annual visit)	0.99 (0.99, 0.99)	0.99 (0.99, 0.99)
Race/ethnicity: non-Hispanic black	1.01 (0.99, 1.02)	1.00 (0.98, 1.02)
Race/ethnicity: Hispanic	1.00 (0.97, 1.02)	0.98 (0.96, 1.01)
Race/ethnicity: other	1.02 (1.00, 1.03)	0.90 (0.86, 0.92)
Dual use status: 50–80% VHA	1.00 (0.99, 1.01)	1.00 (0.99, 1.01)
Dual use status: 50% VHA	0.99 (0.97, 1.00)	1.00 (0.98, 1.01)
Sex: female	0.94 (0.90, 0.99)	0.93 (0.89, 0.98)
Marital status: unmarried	1.07 (1.06, 1.08)	1.07 (1.06, 1.08)
Disability >50%	0.90 (0.89, 0.92)	0.90 (0.89, 0.91)
Smoking status = yes	0.95 (0.94, 0.97)	0.95 (0.94, 0.97)
Statins prescribed: intense vs. regular	0.02 (0.02, 0.02)	0.02 (0.02, 0.02)
HbA1C >8%	1.15 (1.14, 1.17)	—
Acute coronary syndrome	1.04 (1.03, 1.06)	1.05 (1.03, 1.06)
Atherosclerotic cerebrovascular disease	1.04 (1.03, 1.06)	1.04 (1.03, 1.06)
Coronary heart disease	1.18 (1.16, 1.20)	1.18 (1.17, 1.20)
Peripheral artery disease	1.05 (1.04, 1.06)	1.05 (1.04, 1.06)

[a]Per additional comorbidity.
VHA, Veterans Health Administration.
